# Characterization of novel inhibitors of HIV-1 replication that function via alteration of viral RNA processing and rev function

**DOI:** 10.1093/nar/gkt727

**Published:** 2013-08-13

**Authors:** Raymond W. Wong, Ahalya Balachandran, Matthew Haaland, Peter Stoilov, Alan Cochrane

**Affiliations:** ^1^Department of Laboratory Medicine and Pathobiology, University of Toronto, Toronto M5S 1A8, Canada, ^2^Department of Molecular Genetics, University of Toronto, Toronto M5S 1A8, Canada and ^3^Department of Biochemistry, West Virginia University, Morgantown, WV 26506, USA

## Abstract

Expression of the complete HIV-1 genome depends on the appropriate processing of viral RNA. Altering the balance of viral RNA processing impairs replication of the virus. In this report, we characterize two small molecule modulators of HIV-1 RNA processing, 8-azaguanine and 2-(2-(5-nitro-2-thienyl)vinyl)quinoline (5350150), which function by distinct mechanisms to suppress viral gene expression. Although only 8-Azaguanine dramatically decreased accumulation of HIV-1 unspliced and singly spliced RNAs and altered splice site usage, both compounds blocked Gag and Env expression without affecting production of Tat (p16) and Rev regulatory proteins. Subsequent analyses suggest that these compounds affect Rev-mediated RNA transport by different mechanisms. Both compounds induced cytoplasmic accumulation of Rev, suggesting that they function, in part, by impairing Rev function. This conclusion is supported by the determination that both drugs block the nuclear export of genomic HIV-1 RNA to the cytoplasm. Testing confirmed that these compounds suppress HIV-1 expression in T cells at doses below those previously used in humans for tumour chemotherapy. Together, our observations demonstrate that small molecules can be used to inhibit HIV-1 replication by altering another avenue of viral RNA processing, offering the potential for the development of novel therapeutics for controlling this disease.

## INTRODUCTION

The development of highly active anti-retroviral therapies to suppress HIV-1 replication and prevent progression to AIDS has saved countless lives in both the developed and the developing worlds. However, none of the drug combinations in use today provide a cure for this infection, and all necessitate strict adherence to treatment schedules if viral suppression is to be maintained (1,2). In addition, multiple studies have documented the ability of HIV-1 to acquire resistance to drugs in current use and the subsequent transmission of resistant strains of HIV-1 between individuals (3–8). With continued use of anti-retroviral therapies in more diverse populations, it can be anticipated that strains of HIV-1 resistant to current drug cocktails will evolve and become more common (9). As a result, efforts must continue in the search for new anti-HIV compounds that have mechanisms of action distinct from the drugs currently in use.

Current HIV-1 therapeutics target all the enzymatic functions encoded by the virus (reverse transcriptase, protease, and integrase) as well as entry into the cell (10,11). Consequently, novel strategies, to complement the existing therapies, should target other aspects of the virus life cycle. One such step is the processing and expression of HIV-1 RNA. Following integration, a 9-kb transcript is generated from the provirus that is subsequently processed into >40 mRNAs to allow expression of all of the viral structural, enzymatic, and regulatory proteins (12–15). The unspliced (US) 9-kb viral RNA can be used to express the HIV-1 Gag and Gagpol proteins, undergo a single splicing event to produce the singly spliced (SS) class of viral RNAs encoding Vif, Vpr, Vpu and Env, or go through multiple splicing events to generate multiple spliced (MS) RNAs that produce Tat, Rev and Nef. Balancing the extent of HIV-1 RNA splicing is critical to replication fitness because too little splicing results in loss of Tat and Rev, which are essential for enhancing the transcription of the provirus and inducing expression of the viral structural proteins, respectively. In contrast, oversplicing of the viral RNA will impair the production of HIV-1 structural proteins (Gag, Gagpol, and Env), which are essential to new virus assembly (16–18). Because of the need to balance the processing of viral RNA, HIV-1 has evolved various strategies to regulate the extent of splicing as well as the use of specific splice sites, i.e. the presence of suboptimal splicing signals, exon splicing silencers, and exon splicing enhancers (12,15). However, the virus is completely reliant on host splicing factors to carry out the processing of its RNA. Multiple studies during the past decade have demonstrated how changes in abundance of various hnRNP and SR proteins, known regulators of RNA splicing, can dramatically alter the balance between HIV-1 US, SS, and MS RNAs by changing the frequency of specific splice site use, resulting in significant impairment of virus replication (12,15,19–21). The parallel determination that activity of the SR proteins can be modulated by multiple kinases (SRPK1, SRPK2, CLK1, CLK2, CLK3, and CLK4) (22,23) opens up the possibility that modulation of kinase activity could induce changes in HIV-1 RNA splicing and, subsequently, replication. Recent experiments by our group have confirmed this hypothesis by demonstrating that overexpression of specific CLKs results in suppression of viral Gag and Env expression, a response that can be replicated with the use of the small molecule inhibitor, chlorhexidine, a known modulator of CLK function (24).

A recent high-throughput screen of chemical libraries for modulators of SMN2 alternative RNA splicing identified several active compounds (Percifield *et al.*, submitted for publication). To assess whether any of these compounds also altered HIV-1 RNA processing, we tested a subset of these active compounds. In this report, we present data that 8-azaguanine (8-Aza) and 2-[2-(5-nitro-2-thienyl)vinyl]quinoline (designated 5350150) induce a marked decrease in HIV-1 Gag and Env expression, but via distinct mechanisms. The 8-Aza treatment altered viral RNA abundance and use of specific splice sites. In contrast, 5350150 had a more limited effect on HIV-1 RNA processing. Neither compound was found to result in marked changes in the levels of Rev, but both blocked cytoplasmic accumulation of HIV-1 US RNA. Further analysis revealed that these compounds alter Rev subcellular distribution and transport, suggesting that they also impair Rev function. Together, these findings highlight the potential use of small molecules to modulate HIV-1 RNA processing to inhibit viral replication, validating the targeting of this stage of the virus life cycle as a potential therapeutic strategy.

## MATERIALS AND METHODS

For more detailed experimental procedures, see Supplementary Materials and Methods.

### Screening of splice modulator compounds/drugs for effects on HIV-1 RNA processing

Screens were performed using the HeLa rtTA-HIV-Δ*Mls* cell line containing a doxycycline (Dox)-inducible Tet-On HIV-1 LAI strain provirus (25,26) as described in our previous study (24). Compounds tested were obtained from the ChemBridge Online Chemical Store (www.hit2lead.com). Additional tests of compounds were performed using the SupT1-based cell line, 24ST1NELSG, obtained from J. Dougherty (UMDNJ-RWJMS, NJ) (27). With this cell line, latent provirus expression was induced by addition of 1.8 µM phorbol 12-myristate 13-acetate (PMA) after similar treatment procedures described earlier in the text. After ∼24 h, cells were harvested for RNA/protein or XTT assay of cell viability (Sigma-Aldrich, #TOX2), whereas media or cells were lysed with 1% TX-100 for analysis of HIV-1 gene expression by p24^CA^ ELISA described later in the text. For experiments using infected patient samples, peripheral blood mononuclear cells (PBMCs) were first depleted of CD8+ T cells using Dynabeads CD8 (Invitrogen, #111.47D) as outlined by manufacturer. Depletion was confirmed by flow cytometry analysis of CD8 and CD3 surface markers. Remaining cells were then activated by treatment with anti-CD3 and anti-CD28 antibodies (Bio Legend #302914 and 317304, respectively; 1 µg/ml of each) as well as 50 U/ml of IL-2 (BD Pharmingen #554603) in the presence or absence of indicated drugs. Media (0.5 ml) were collected every 3–4 days and replaced with fresh media (0.5 ml) containing 20 U/ml of IL-2 and fresh drug. Effect of compounds on cell viability was monitored in parallel by XTT assay and expressed relative to control (DMSO)-treated cells. HIV-1 growth in cultures was monitored by p24^CA^ ELISA of cell supernatants.

### Analysis of HIV-1 expression

To monitor HIV-1 gene expression, cell culture supernatants or cell lysates were assayed for production of Gag protein by a HIV-1 p24^CA^ antigen capture assay kit (AIDS & Cancer Virus Program, NCI-Frederick, Frederick, MD, USA). For further analysis of HIV-1 protein expression, western blots were performed on cell lysates. The antibodies and conditions used for Tat, anti-tubulin and isotype-specific HRP-conjugated antibodies were used as described (24). Additional antibodies used include anti-p24 from hybridoma 183 and mouse anti-gp120 from hybridoma 902 (AIDS Research and Reference Reagent Program, Division of AIDS, NIAID, NIH), mouse anti-Rev (Abcam, #ab85529) and rabbit anti-GAPDH (Sigma, #9545). Generally, Western Lightning-ECL (Perkin-Elmer, #NEL101) or Western Lightning Plus-ECL (#NEL105) for anti-Rev, -Tat and -gp120 blots was used. Signals were acquired by a ChemiDoc MP with Image Lab software (Bio-Rad) or exposed onto autoradiography film.

### Effect of compounds on RNA processing and transport

RNA was extracted from cells, reverse transcribed and HIV-1 mRNA levels quantitated by qRT-PCR as described (24). The effect of drugs on HIV-1 splice site usage within the 2-kb MS RNA class was analysed by RT-PCR of cDNA as previously described (24). For analysis of alternative splicing of cellular RNAs, the inclusion levels of 157 alternatively spliced exons and splice sites located in 96 alternatively spliced regions of 85 genes (Supplementary Dataset S1) was assayed by medium throughput RT-PCR. This set of events had been previously suggested to be linked to cell transformation and was available for use in the laboratory. Labelled PCR products were denatured in formamide and quantified using an ABI Prism capillary sequencer (Life Technologies). The inclusion level of each exon was calculated as the amount of transcripts carrying the alternative exon relative to the total amount of all transcripts detected in the PCR reaction.

To assess the impact of the various compounds on HIV-1 US RNA subcellular distribution, cells were treated with compounds as outlined earlier, processed and probed with Stellaris™ FISH probes comprising of a mixture of 48 Quasar 570-labelled 20-mer oligonucleotides spanning the HIV-1 Gag coding region as outlined by the manufacturer (Biosearch Technologies). Images were captured by a Leica DMR epifluorescence microscope at 630 × magnification.

### Effect of compounds on HIV-1 Rev subcellular distribution

HeLa Rev cells (stably expressing HIV-1 Rev) were treated with compounds as indicated. For studies using import or export inhibitors, leptomycin B (LB) was added at 20 ng/ml for 2 h before fixing, whereas cells treated with 4 µg/ml actinomycin D were incubated for 2 h before fixation. Slides were processed as detailed in the Supplementary Materials and Methods and probed by primary rabbit antibody against Rev, detected by either FITC- or Cy5-labelled donkey anti-rabbit antibody (Jackson Laboratories), and imaged at 630× or 400× magnification by epifluorescence microscopy. To examine the effects of drugs on the subcellular localization of host nuclear proteins, fixed cells were stained with mouse anti-hnRNP A1 (from Benoit Chabot), anti-SRp20 (Invitrogen, # 334200), or anti-SC35 (Sigma, # S4045) antibody.

### Statistical analysis

Data were analysed by Microsoft Excel and expressed as means ± standard error of the mean (SEM). Differences between two groups of data [compound/drug treatment versus DMSO (+Dox) control or compound/drug treatment versus DMSO (+HIV) control] were compared by two-tailed Student’s *t*-test. Statistical significance of results is indicated on graphs as follows: *P* < 0.05, *, *P* < 0.01, ** and *P* < 0.001, ***, unless otherwise indicated.

## RESULTS

### 8-Aza and 5350150 suppress HIV-1 structural protein expression

**Figure 1.**
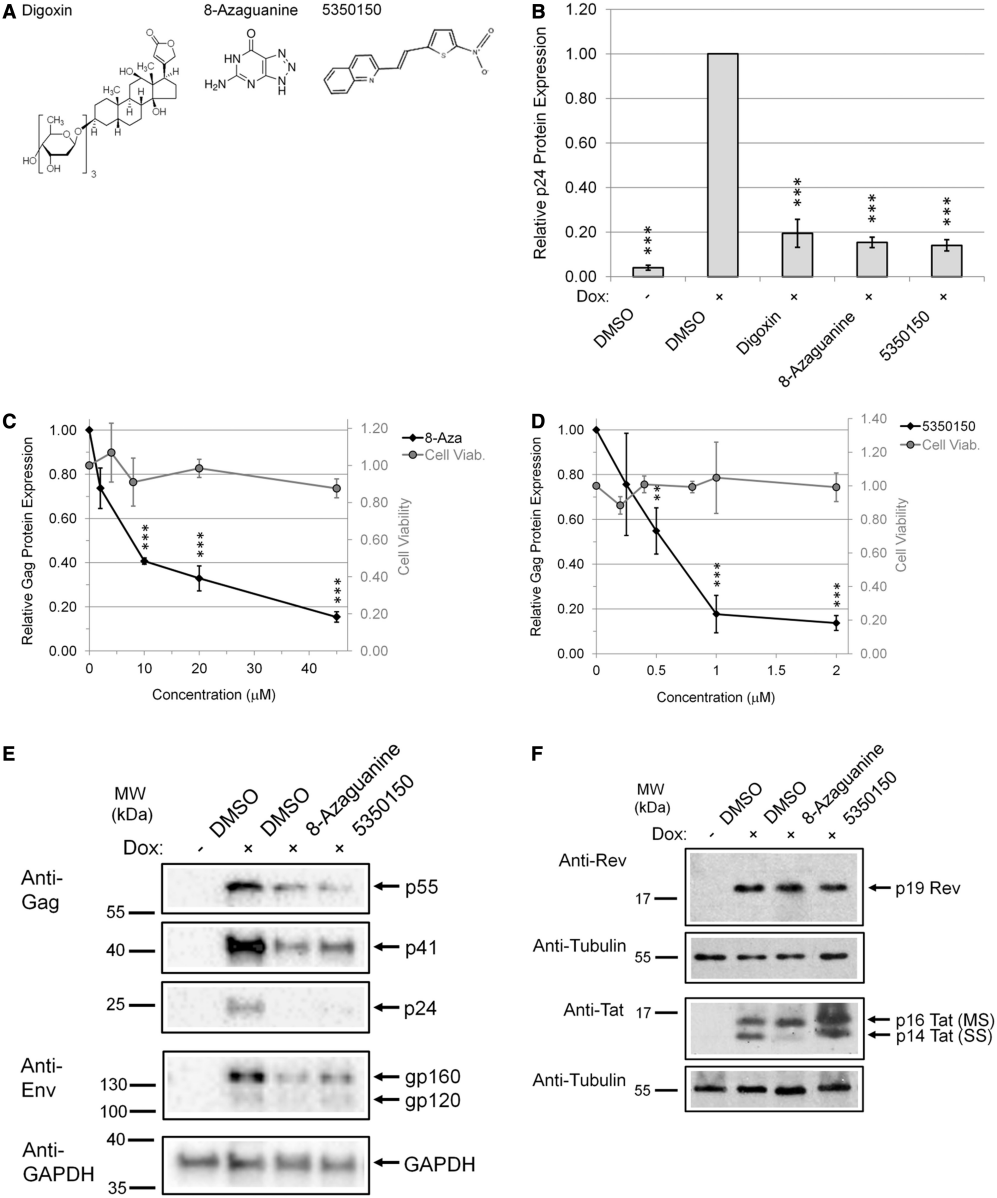
Identification of 8-Aza and 5350150 as potent inhibitors of HIV-1 gene expression. HeLa rtTA-HIV-ΔMls cells were treated with (**A**) indicated compounds (80 nM digoxin, 45 µM 8-Aza, and 2 µM 5350150) or DMSO (control) for 4 h before induction of viral gene expression with (+) and without (−) Dox for 20 h. Equal concentrations of DMSO solvent were present in each treatment. (**B**) Cell culture supernatants were harvested after compound treatments for analysis of HIV-1 Gag protein expression by p24^CA^ ELISA. For information on the relative efficacy of each compound, dose–response curves of the inhibition of HIV-1 gene expression by (**C**) 8-Aza (0–45 µM) and (**D**) 5350150 (0–2 µM) were generated (black diamonds). In parallel, the effect of compounds on cell viability was measured by XTT assay (gray circles). Data were averaged from *n* ≥ 5 independent experiments in (B) and *n* ≥ 4 in (C and D), displayed as a fraction relative to DMSO (+) control, error bars are SEM, and statistical significant changes [between compound treatments from DMSO (+) control] indicated by asterisks as detailed in ‘Materials and Methods’ section. In addition, cells extracts were analysed by western blot with antibodies for (**E**) HIV-1 structural proteins, Gag and Env, and (**F**) viral regulatory factors, Rev and Tat. Blots were probed with either anti-GAPDH or anti-tubulin antibody to confirm equal loading of protein samples. Data are representative of *n* ≥ 3 experiments in (E) and *n* ≥ 6 in (F). Lanes were cropped and assembled from the same blots.

To evaluate a panel of RNA splicing modulators for their effect on HIV-1 RNA processing, we performed initial analyses using the HeLa rtTA-HIV-Δ*Mls* cell line described previously (24). In brief, the cell line contains an HIV-1 LAI provirus in which the RT and IN reading frames had been deleted, the Nef region replaced with the rtTA activator, and multiple TetO operators inserted into the U3 region (25,26). These modifications render expression of the provirus dependent on addition of doxycycline (Dox) to the media. Cells were treated for 4 h with each compound (Figure 1A) at the doses indicated, and then viral expression was induced by Dox addition for ∼20 h. Media and cells were harvested at 24 h and analysed for alterations in HIV-1 gene expression. Analysis of cell media from various treatments for HIV-1 p24 Gag protein (Figure 1B) revealed that addition of either 8-Aza or 5350150 resulted in substantial reduction in Gag expression, comparable with that seen in the absence of Dox, with maximum suppression requiring ∼20–45 µM 8-Aza or ∼1–2 µM 5350150 (Figure 1C and D). Parallel assays of the effects of the compounds on cell viability (XTT assay, Figure 1C and D) determined that neither 8-Aza nor 5350150 resulted in marked reductions in viability for the time frame or drug concentrations used.

To probe the basis of the response seen, the effects of either compound on the expression of HIV-1 Gag, Env, Tat, or Rev were analysed by western blot. As shown in Figure 1E, western blots of cell lysates determined that both 8-Aza and 5350150 reduced HIV-1 Env expression to a similar extent as Gag. However, neither compound had much effect on the expression of other HIV-1 proteins such as Rev and p16 Tat encoded by MS RNA (Figure 1F) in contrast to another modulator of HIV-1 RNA processing, chlorhexidine (24). These findings support the conclusion that general protein expression is not affected by these compounds. Treatment with 8-Aza caused depletion of p14 Tat, a Tat isoform encoded by SS RNA that is dependent on Rev-mediated export, suggesting that at least one of these compounds may act by impairing Rev function.

### 8-Aza dramatically alters HIV-1 RNA processing

**Figure 2.**
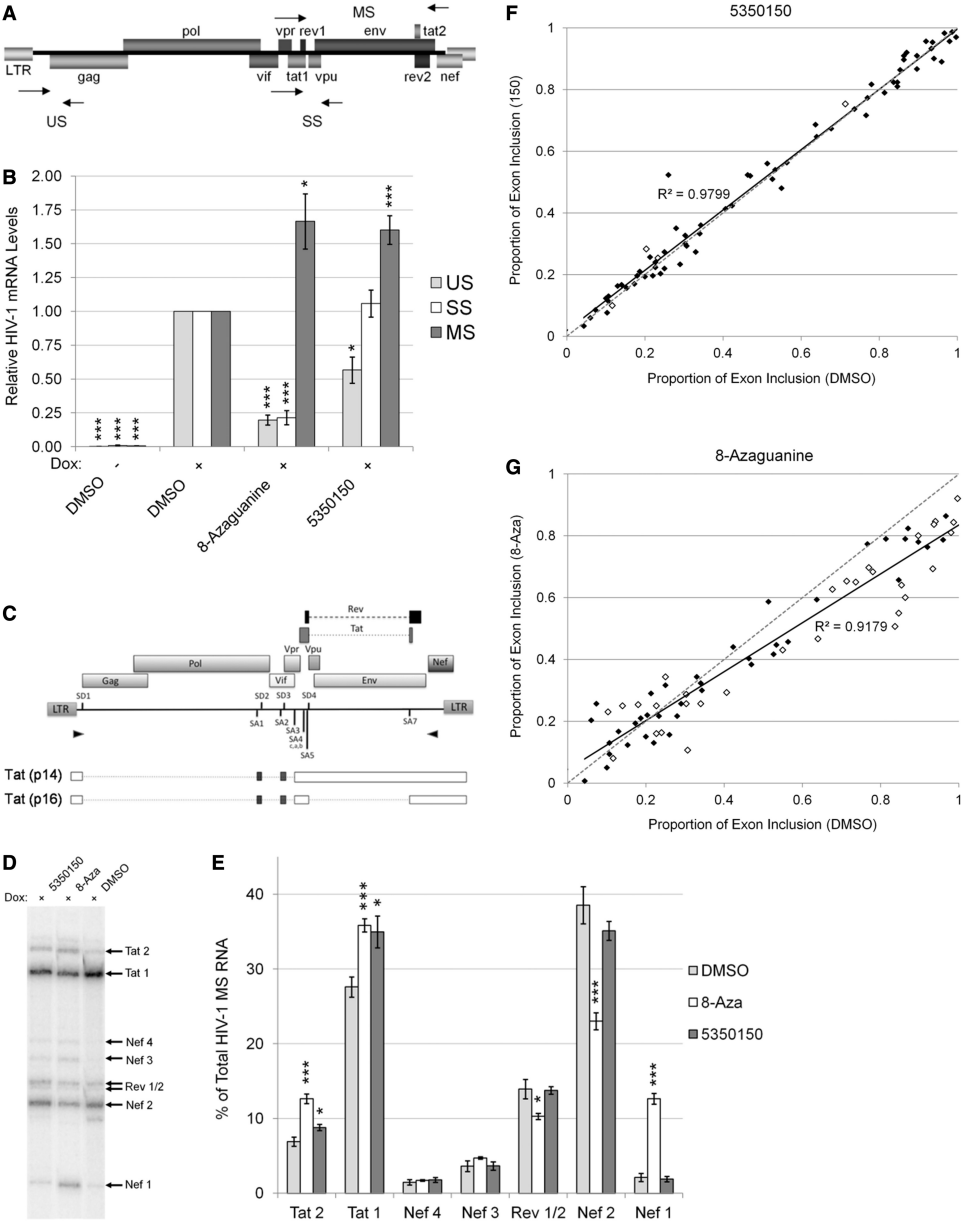
Effect of 8-Aza and 5350150 on HIV-1 and host RNA processing. The effects of 8-Aza or 5350150 on HIV-1 mRNA levels were assessed by qRT-PCR (as described in ‘Materials and Methods’ section) of HeLa rtTA-HIV-Δ*Mls* cells treated with 45 µM 8-Aza, 2 µM 5350150 or DMSO (control) for 24 h with doxycycline before harvest. (**A**) The position of the primers (arrow points) used in qRT-PCR. (**B**) Abundance of HIV-1 unspliced (US, gray), singly spliced (SS, white), and multiply spliced (MS, black) mRNAs are shown relative to DMSO (+) controls. The housekeeping gene β-actin served as an internal loading control for the normalization of these data. Shown are data averaged from ≥ 5 experiments, error bars are SEM, and significant changes from control indicated by asterisks as described in ‘Materials and Methods’ section. (**C-E**) Changes in HIV-1 splice site use were assayed by RT-PCR of the 2 kb, MS class of HIV-1 mRNA (as outlined in ‘Materials and Methods’ section). (C) The position of the primers (arrow points) used to amplify MS mRNA species. (D) Representative RT-PCR gel of the levels of each MS mRNA species (arrows) from HeLa rtTA-HIV-Δ*Mls* cells treated with doxycycline and 45 µM 8-Aza, 2 µM 5350150 or DMSO as performed in Figure 1. See Supplementary Figure S1 for a description of the PCR products generated. (E) Graph summarizing the effects of 8-Aza (white), 5350150 (black), and control treatments (gray) on the level of each MS mRNA species (*x*-axis) relative to the total HIV-1 MS mRNA (*y*-axis), displayed as percentage (%) of the total HIV-1 MS RNA. Data were averaged from ≥ 6 experiments, error bars are SEM, and significant differences from control indicated by asterisks as described in ‘Materials and Methods’ section. (**F** and **G**) RNAs from treatments outlined earlier in the text were subsequently used to analyse for changes in host RNA splicing. Primer pairs spanning host RNA alternative splicing event were used to amplify from cDNA and amplicons quantitated following resolution by capillary electrophoresis. Shown is a comparison of the level of alternative exon inclusion following treatment with (F) 5350150 or (G) 8-Aza relative to DMSO-treated cells. Values are the average of three independent trials, and events significantly different from control (DMSO) at *P* < 0.05 are indicated by open diamonds. The dotted line (grey) reflects the anticipated results if the drugs had no effect on host RNA splicing. The solid line (black) is linear regression of the data with strength of the correlation indicated.

Based on the effect of these compounds on HIV-1 gene expression and recently reported modification of RNA splicing (Percifield *et al.*, unpublished data), we examined whether either compound altered HIV-1 RNA accumulation or splice site selection. As indicated in Figure 2A and B, qRT-PCR analyses determined that 8-Aza treatment significantly altered HIV-1 RNA accumulation (similar to chlorhexidine) (24). Treatment with this compound reduced both US and SS viral RNA abundance to ∼20% of control (+Dox) levels, whereas MS RNA accumulation was increased by ∼1.5-fold. In contrast, 5350150 treatment led to only modest changes, US RNA being reduced to ∼60% of control, no effect on SS RNA accumulation, and a ∼1.5-fold increase in MS RNA abundance. In addition, there were no significant effects on the overall RNA concentration or copies of the housekeeping gene, actin (data not shown). In light of these alterations, we also investigated whether either compound elicited any changes in the frequency of splice site use. To this end, RT-PCR was performed to amplify viral MS RNAs, and the products were analysed by separation on denaturing gels. As seen in Figure 2C–E, treatment with 5350150 had little effect on the frequency of splice site usage within MS viral RNAs. In contrast, 8-Aza induced a marked alteration in splice site usage, strongly promoting the generation of Nef1 product [generated by the joining of the first 5′splice site of HIV (SD1) with the last 3′splice site (SA7)] with a comparable reduction in Nef2 RNA. In addition, there was a modest reduction in the level of Rev1/2 and increase of Tat1 and Tat2 RNAs.

### Measuring the effect of 8-Aza and 5350150 on host cell RNA processing

The changes in HIV-1 RNA processing in response to either 8-Aza or 5350150 raised the question as to whether either compound had much broader effects on RNA splicing. To address this point, we analysed for changes in ∼90 known alternative splicing events on addition of either compound (see Supplementary Dataset S1 for the list of genes analysed and primers used). Primer pairs spanning the alternative splicing events were used to amplify cDNA prepared from mock or treated cells and changes in exon inclusion assessed by quantitation of the amplicons following separation by capillary eletrophoresis. As shown in Figure 2F (see Supplementary Dataset S2 for values obtained for individual genes), comparison of the level of alternative spliced products in the presence and absence of drug revealed few significant differences from control in the case of 5350150. In contrast, treatment with 8-Aza was observed to induce significant alterations in a subset of alternative splicing events (Figure 2G). The majority of the affected RNAs showed reduced exon inclusion in response to this drug but none to the same extent as seen for HIV-1 RNA. Based on these findings, it can be concluded that neither 5350150 nor 8-Aza is acting through a general perturbation of the splicing process but rather are having selective effects on a subset of host RNAs.

### Effects of 8-Aza and 5350150 on HIV-1 expression in CD4+ T cells

**Figure 3.**
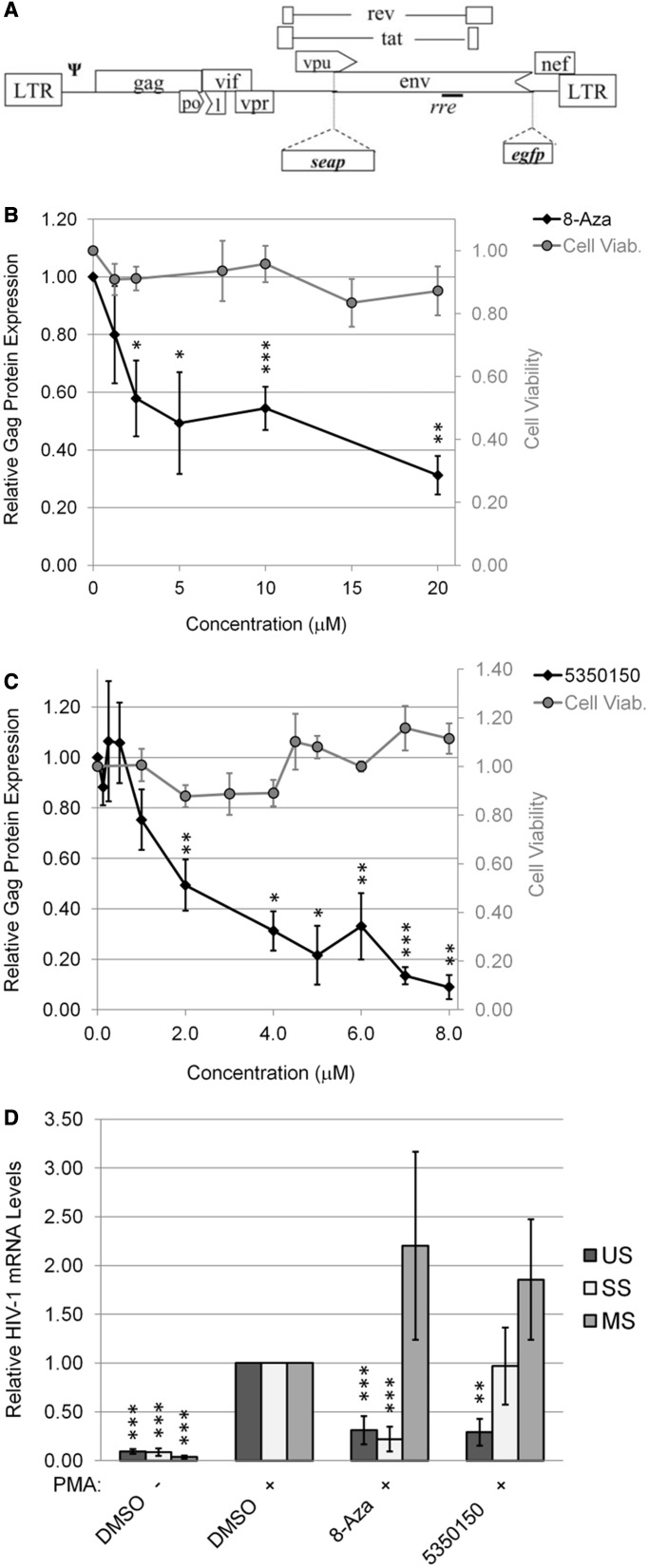
Both 8-Aza and 5350150 inhibit HIV-1 expression in a CD4+ T cell line. Using a CD4+ T cell line (SupT1) stably transduced with an HIV-1 provirus [(**A**) 24ST1NLESG], cells were treated with (**B**) 8-Aza or (**C**) 5350150 at concentrations indicated, and HIV-1 provirus expression induced by addition of PMA after 4 h as detailed in ‘Materials and Methods’ section. Equal concentrations of DMSO solvent were present at each concentration tested. After 24 h, media were collected and the levels of HIV-1 gene expression assessed by p24^CA^ ELISA. Cell viability was also assessed in parallel by XTT assays. (**D**) Effect of 8-Aza and 5350150 on HIV-1 mRNA accumulation. Following treatment of cells with DMSO, 10 µM 8-Aza or 7 µM 5350150, virus gene expression was induced by addition of PMA. After 24 h, total RNA was extracted and viral RNA abundance analysed by qRT-PCR as outlined in ‘Materials and Methods’ section. Shown are data averaged from ≥3 independent experiments, error bars are SEM and significant changes from control indicated by asterisks as described in ‘Materials and Methods’ section.

Having demonstrated the capacity of 8-Aza and 5350150 to suppress HIV-1 gene expression in our experimental system, we validated their anti-viral activity in a human CD4+ T cell line (24ST1NLESG) containing an inducible HIV-1 provirus (Figure 3A) (27). As shown in Figure 3B–C, both 8-Aza and 5350150 resulted in a marked reduction in HIV-1 Gag expression without significant effects on cell viability at all concentrations shown. Additional western blots confirmed that, although these compounds reduced Gag expression, they had little to no effect on Tat or Rev levels in this cell line (Supplementary Figure S2). Subsequent analysis of the effect of the compounds on viral RNA accumulation (Figure 3D) revealed that each had effects similar to those seen in the HeLa rtTA-HIV-Δ*Mls* cell line; 8-Aza reduced the level of both US and SS RNAs by 80%, whereas 5350150 decreased only US RNA levels with no or only moderate effects on SS and MS RNA abundance. The similarity in effect of both 8-Aza and 5350150 in two cell lines examined suggests that the targets of these two compounds are conserved.

**Figure 4.**
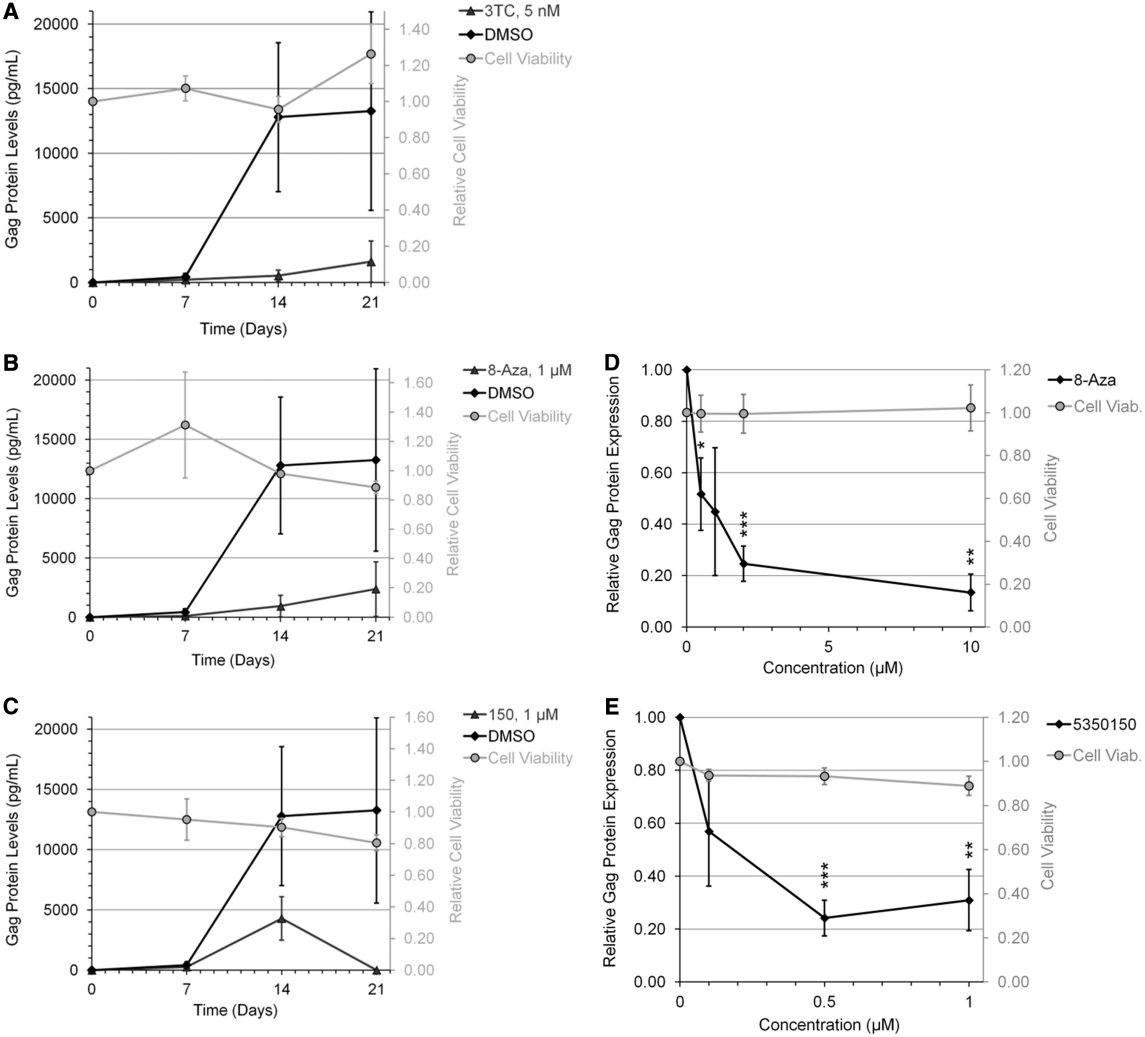
Suppression of HIV-1 replication in chronically infected PBMCs by 8-Aza and 5350150. CD8-depleted PBMCs from chronically HIV-1-infected patients were activated by treatment with anti-CD3 and anti-CD28 antibodies to induce HIV-1 growth in the culture. Cells were treated with 3-TC, 8-Aza, or 5350150 (triangles), or control (DMSO, diamonds). Equal concentrations of DMSO solvent were present at each concentration of compound/drug tested. Media were harvested at multiple times points (0-21 days) to assess virus replication by p24^CA^ ELISA of Gag. Shown (**A**, **B**, **C**) are the viral growth curves using 5 nM 3-TC, 1 µM 8-Aza or 1 µM 5350150, and (**D**, **E**) the dose response effects of the indicated drugs after 14 days in culture. Results shown are derived from assays performed on four different patient samples. Effect of drugs on cell viability (Cell Viab.) was also tested by XTT assay and results expressed relative to DMSO-treated cells (grey circles). All error bars are SEM and statistical significance indicated by asterisks as detailed in ‘Materials and Methods’ section.

To validate our findings in a more relevant setting, the ability of 8-Aza or 5350150 to suppress HIV-1 replication was examined in the context of CD8-depleted PBMCs obtained from treatment-naïve HIV-infected patients. Cells were incubated in the presence of lamivudine (3-TC) (a reverse transcriptase inhibitor), 8-Aza or 5350150 and media harvested at various days after cell activation. As shown in Figure 4A–C, both 8-Aza and 5350150 displayed significant suppression of viral growth at a dose of 1 µM. Subsequent dose response analysis (Figure 4D and E) determined that optimal suppression of HIV-1 growth was obtained at 10 µM of 8-Aza or 0.5 µM of 5350150.

### Both 8-Aza and 5350150 alter Rev localization

**Figure 5.**
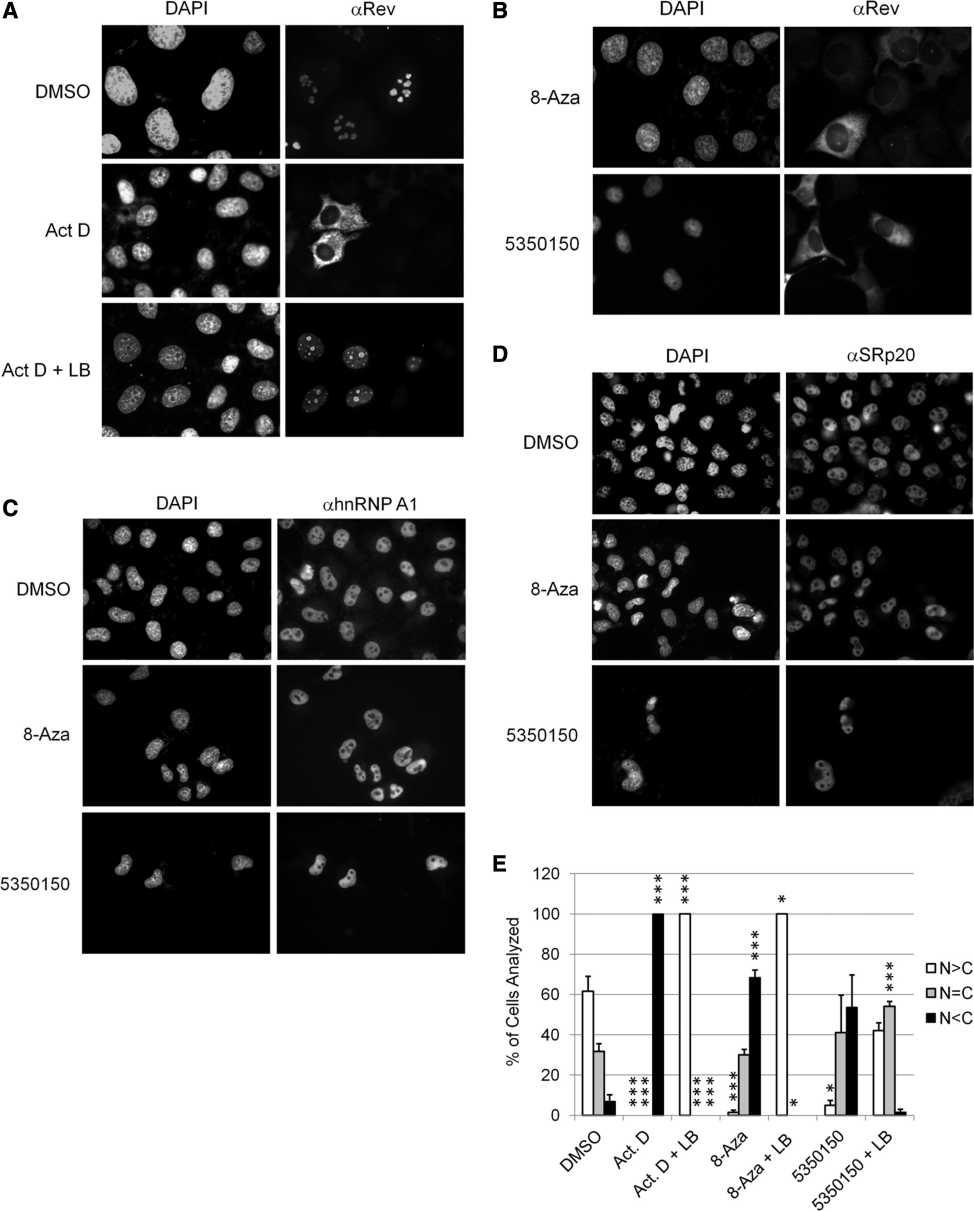
Both 8-Aza and 5350150 alter the subcellular distribution of HIV-1 Rev but not nuclear proteins of the host cell. HeLa Rev cells stably expressing HIV-1 Rev were (**A**) control treated (DMSO) or incubated with 4 µg/ml of Act. D with (+) or without LB for 2 h or (**B**) treated with compounds overnight with 45 µM 8-Aza or 2 µM 5350150 as described in Figure 1. After fixation and permeabilization, localization of Rev was detected by immunofluorescence using a rabbit anti-Rev antibody followed by either a FITC- or Cy5-conjugated anti-rabbit IgG antibody. In parallel, cells were stained with antibodies for (**C**) hnRNP A1 or (**D**) SRp20 as detailed in ‘Materials and Methods’ section. Shown are representative images (gray scale) from three independent trials. Cells were stained with DAPI to allow imaging of the nuclei. Magnification 400×. (**E**) To determine whether 8-Aza or 5350150 were altering Rev localization by affecting Rev import, HeLa Rev cells were treated with DMSO, 2 h with Act. D (as a control that induces Rev cytoplasmic accumulation) or overnight with either 45 µM 8-Aza or 2 µM 5350150, and then left untreated or treated with LB (+LB) for 2 h before fixation. Rev distribution was assessed in individual cells by immunofluorescent staining using a rabbit anti-Rev antibody as detailed in ‘Materials and Methods’ section. Individual cells were scored as having predominately nuclear (N > C, white), whole cell (N = C, grey) or predominately cytoplasmic (N < C, black) patterns of Rev distribution. Shown are data averaged from three independent experiments, error bars are SEM and significant changes from control indicated by asterisks as described in ‘Materials and Methods’ section. Representative images of the distribution patterns observed are provided in Supplementary Figure S3C.

Although the alteration in HIV-1 US, SS, and MS RNAs induced by 8-Aza could account for the reduced expression of the corresponding proteins (Gag and Env), a similar case cannot be made for 5350150. The loss of both Gag and Env expression on addition of 5350150 suggested that it might impair Rev function. The ability of Rev to shuttle between the nucleus and cytoplasm is critical for its ability to export US and SS viral RNAs to the cytoplasm and their subsequent translation (28,29). Consequently, alterations in either Rev nuclear import or export could reduce its function and be reflected in changes in its subcellular distribution or localization of Rev-dependent viral RNAs. As a test of this hypothesis, the effect of 8-Aza or 5350150 on Rev subcellular distribution was examined by immunofluorescence microscopy. To monitor changes in Rev localization in response to drugs, we used a HeLa Rev cell line that constitutively expresses Rev alone at higher levels than the HeLa rtTA-HIV-Δ*Mls* cell line to better assess changes in Rev activity. As previously reported and shown in Figure 5A, Rev is predominately localized to the nucleus and, in particular, the nucleolus in the absence of any treatment (DMSO) (30). However, on addition of actinomycin D (Act. D), Rev accumulates in the cytoplasm (31). This response can be reversed by leptomycin B (LB, an inhibitor of the Rev export mediator, Crm1) (32). Act. D acts to invert the relative rates of Rev import into and export out of the nucleus, resulting in export being the dominant pathway. LB, which inhibits Rev export by inactivating Crm1, induces nuclear accumulation of Rev by allowing only import to occur. To assess whether either 8-Aza or 5350150 also affects Rev transport, cells were treated with either compound overnight. As shown in Figure 5B, addition of either 8-Aza or 5350150 induced Rev accumulation in the cytoplasm (similar to the effect on Act. D treatment). Subsequent time course experiments determined that 8-Aza induced a shift in Rev subcellular distribution within 4 h, whereas the same effect required ∼16 h of treatment with 5350150 (Supplementary Figure S3). To determine whether the effects of 8-Aza or 5350150 on Rev subcellular distribution reflected a general perturbation in nuclear protein distribution, we also examined for changes in SC35 as well as known shuttling factors, hnRNP A1 and SRp20 (33,34). As shown in Figure 5C and D and Supplementary Figure S4, neither 8-Aza nor 5350150 induced any detectable alteration in distribution of other nuclear or nuclear shuttling proteins examined, suggesting that the responses seen for Rev are selective.

Changes in Rev localization could reflect changes in one or both of the rates of Rev nuclear export or import. To gain insight into which process is affected, we assessed the ability of LB to induce nuclear accumulation of Rev in the presence of compound (as described in Figure 5A, where LB reversed the effects of Act. D-treated cells). Cells were treated with either 8-Aza or 5350150 overnight to induce Rev relocalization, and then treated with LB for several hours before fixation and analysis. As shown in Figure 5E and Supplementary Figure S3C, LB treatment was successful in reversing the effect of 8-Aza and Act. D on Rev subcellular distribution, inducing complete nuclear accumulation of Rev. Thus, 8-Aza is not solely impeding Rev nuclear import. In contrast, LB treatment only partially induced nuclear accumulation of Rev in the presence of 5350150, indicative of reduced rate of Rev nuclear import. The differences in response of 8-Aza and 5350150 to LB add further evidence that these compounds function via distinct mechanisms.

**Figure 6.**
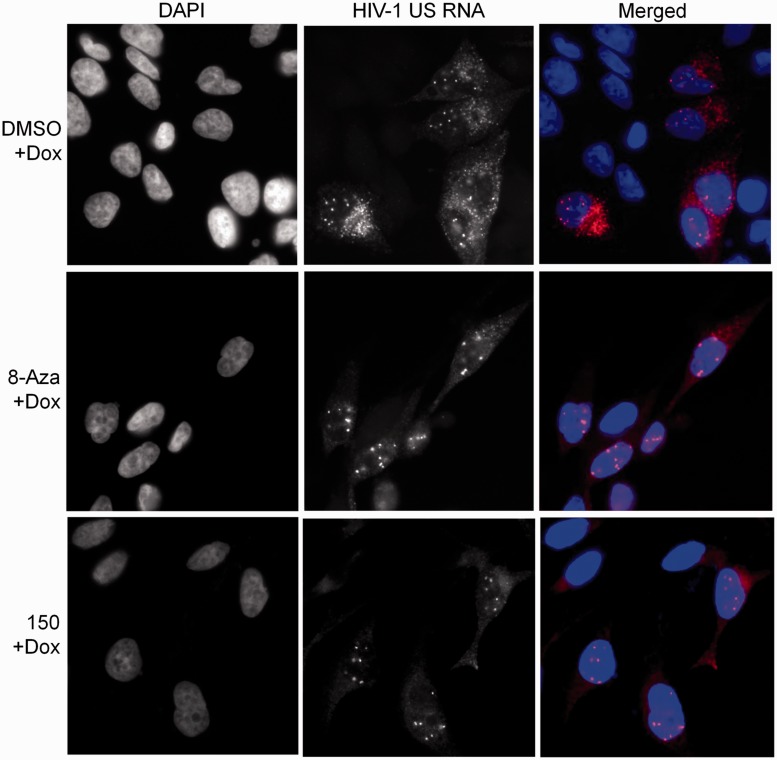
8-Aza and 5350150 inhibit the transport of HIV-1 US RNA to the cytoplasm. To assess the effect of compound treatments on the subcellular distribution of HIV-1 genomic US RNA, HeLa rtTA-HIV-Δ*Mls* cells were treated overnight with DMSO (DMSO + Dox), 40 µM 8-Aza + Dox or 2 µM 5350150 (150 + Dox) for 4 h before induction of viral gene expression with Dox for 20 h as described in Figure 1. Cells were subsequently fixed and processed for *in situ* hybridization of HIV-1 US RNA as detailed in ‘Materials and Methods’ section. Shown are representative distribution patterns observed from each treatment. Cells were stained with DAPI to detect nuclei (left), HIV-1 US RNA was detected in the Cy3 channel (middle) and images were merged in colour (right). Patterns shown for each treatment are representative of *n* ≥ 4 independent experiments.

To assess whether alterations in Rev localization induced by either compound affected HIV-1 RNA transport, *in situ* hybridization for HIV-1 US RNA was performed. As shown in Figure 6, HIV-1 US RNA (DMSO + Dox) is normally distributed throughout both the nucleus and cytoplasm; the discrete spots in the nucleus reflecting accumulation at the sites of provirus transcription. However, treatment with either 5350150 or 8-Aza resulted in the detection of HIV-1 US RNA only in discrete foci in the nucleus with little signal in the cytoplasm, consistent with a block in the nuclear export of this RNA.

## DISCUSSION

The development of highly active anti-retroviral therapies has saved countless lives because of its ability to suppress virus replication, offering the prospect of a normal lifespan for those infected with HIV-1. However, it is not a cure (2). Further complicating treatment is the capacity of HIV-1 to rapidly adapt to both immune and drug pressure. Such evolution is evident in the transmission of HIV-1 strains resistant to one or more drugs (1,4,5,7). To ensure that patients continue to have a range of treatment options, efforts should be directed towards the development of novel therapeutics that complement the action of existing drugs. To this end, we have been exploring the use of small molecular modulators of alternative RNA splicing to suppress HIV-1 replication. In support of the viability of this approach, recent work by another group has used indole derivatives to successfully inhibit HIV-1 RNA processing (21,35). In this report, we demonstrate that both 8-Aza and 5350150 are potent suppressors of HIV-1 gene expression, each operating by different mechanisms and distinct from that previously documented for chlorhexidine (24).

In the 1950s, 8-Aza was tested in humans as a chemotherapeutic agent for the treatment of leukaemia at doses between 200 and 1000 mg (∼0.25–1.3 mM). Although some anti-tumour responses were observed, undesirable reactions at these doses limited its therapeutic use (36,37). Early studies in HeLa cells indicated only minimal reduction in cell growth rates at doses of <40 µM 8-Aza (38). At doses of 300 µM, there was not only a small (∼10%) reduction in RNA synthesis but also a ∼50% decrease in total protein production in these cells (38). These observations were consistent with 8-Aza predominately affecting translation (39), in particular initiation of translation, as confirmed in subsequent studies (40). On the other hand, parallel analysis of 8-Aza in two *in vitro* translation systems (where protein production was not dependent on RNA synthesis) failed to show any change in protein synthesis, supporting the conclusion that 8-Aza does not act directly on protein synthesis but rather acts by altering the mRNA template (41,42). In addition, other studies of this anti-metabolite confirmed that it was incorporated in RNA but had little to no effect on ribosomal RNA processing (43), suggesting that it was acting at the level of mRNA. More recent studies have identified additional effects of 8-Aza, including selective changes in CD26 expression (44) and Von Hippel–Lindau protein stability (45). Our study of this compound expands its range of effects. The lack of any alteration in Rev or p16 Tat levels in the presence of 8-Aza indicates that the concentration required to suppress HIV-1 structural protein expression has limited effect on total protein synthesis. Rather, at ∼45 uM, 8-Aza induced significant alterations in viral RNA splicing as well as a block in viral RNA export because of a shift in Rev subcellular distribution.

**Figure 7.**
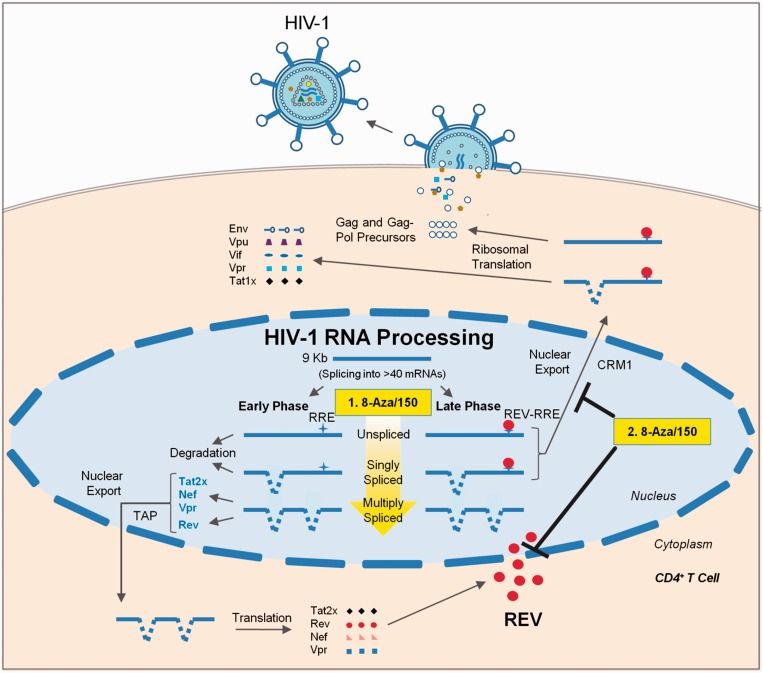
8-Aza and 5350150 inhibit HIV-1 replication by altering viral RNA processing. This diagram outlines the stages of RNA processing during the HIV-1 life cycle. This study demonstrates that 8-Aza and 5350150 are potent inhibitors of HIV-1 replication that perturb viral RNA processing, a stage of the virus life cycle not targeted by current anti-retroviral therapies. 8-Aza and 5350150 inhibit the virus by altering specific pre-mRNA splicing events under the control/influence of the host. This includes (i) inducing oversplicing of HIV-1 pre-mRNA (depicted by an increasingly yellow arrow), which reduces both US and SS mRNA levels (with the exception of 5350150 on SS mRNA), and (ii) altering the localization of HIV-1 Rev, resulting in sequestration of incompletely spliced (US and SS) RNAs to the cell nucleus. Both of these mechanisms lead to a reduction in US and SS viral mRNAs available for translation, perturbing the synthesis of a subset of HIV-1 regulatory and accessory factors as well as structural proteins/enzymes necessary for new virion assembly and infection. This study confirms that small molecule inhibitors that alter viral RNA processing can potently inhibit HIV-1 replication, signifying the potential of directing novel therapeutics to this stage for control of this disease.

In the case of splicing, treatment with 8-Aza resulted in a significant reduction in accumulation of the incompletely spliced (US and SS) viral RNAs with a slight increase in MS viral RNA levels, consistent with induction of oversplicing or a reduced stability of the incompletely spliced viral RNAs. In addition, we detected a shift in the pattern of splice site usage within the viral MS RNAs with a dramatic increase in the generation of Nef1 RNA, corresponding to the joining of the first 5′ss of HIV-1 (SD1) to the last 3′ss (SA7), and a reduction in Nef2 RNA levels. Analysis of the effect of 8-Aza on host RNA splicing revealed some perturbations, but only to a limited number of the splicing events analysed and none to the same extent as observed for HIV-1. In light of the known incorporation of 8-Aza into RNA, it may be acting to alter recognition of splicing regulatory sequences by host factors. Consistent with this possibility, mutational inactivation of the purine-rich GAR exon splicing enhancer adjacent to the major 5′ss of Env (SD4) results in increased accumulation of Nef1 similar to the effect seen with 8-Aza treatment (46). Alternatively, incorporation into hnRNP A1 binding sites (consensus UAGGG) throughout the viral RNA may interfere with the ability of hnRNP A1 to suppress HIV-1 splice site usage (15,47), potentially explaining the oversplicing phenotype observed. Although the incorporation of 8-Aza into RNA could explain some of the effects on viral RNA processing, the mechanism underlying its effect on Rev function is less apparent. The rapid shift in the subcellular distribution of Rev on 8-Aza addition, coupled with the block in accumulation of HIV-1 US RNA in the cytoplasm, is consistent with an inhibition of Rev function. The ability of LB treatment to reverse the effect of 8-Aza on Rev localization indicates that this compound does not prevent Rev shuttling but rather alters the relative rates of Rev nuclear import and export, from favouring nuclear import to preferring nuclear export. However, the inability of 8-Aza to alter the subcellular distribution of other known shuttling proteins (hnRNP A1 and SRp20) indicates that its mechanism is likely selective for factors regulating Rev movement. Taken together, the data (summarized in Figure 7) suggest that 8-Aza functions to suppress HIV-1 structural gene expression by (1) inducing oversplicing or altering stability of the viral RNA to reduce the amount of US and SS RNA available for export/translation and (2) reduce the level of Rev in the nucleus, impairing the transport of the US and SS RNAs to the cytoplasm. The determination that 8-Aza is also able to suppress HIV-1 expression in the context of a T cell line and PBMCs indicates that it is affecting a common pathway across multiple cell lines.

The suppression of HIV-1 gene expression by 5350150 appears to occur through a distinct mechanism than 8-Aza. The 5350150 addition resulted in more limited alterations in abundance of the various HIV-1 RNAs and no significant shift in splice site usage. Assays measuring the effects of 5350150 on host alternative RNA splicing did not detect any marked changes, indicating that the compound is acting in a highly selective manner. Subsequent analyses indicate that 5350150 suppresses HIV-1 structural protein (Gag and Env) expression by affecting Rev function, as evidenced by the shift in Rev subcellular distribution and the absence of HIV-1 US RNA in the cytoplasm on treatment (Figure 7). Furthermore, 5350150 induces these alterations in a fashion distinct from either 8-Aza or Act. D as suggested by the slower kinetics of Rev relocalization to the cytoplasm and the failure of LB to fully induce nuclear accumulation of Rev. The latter observation suggests that 5350150 may be acting to impair components of Rev nuclear import. However, the slow kinetics of the change in Rev localization on addition of 5350150 raises the possibility that the effect seen is indirect, mediated by altering the abundance of factors regulating Rev movement. As with 8-Aza, the absence of any alteration in distribution of other shuttling proteins (hnRNP A1 or SRp20) suggests that the net effect of 5350150 is likely selective to Rev. Analysis of structural variants of 5350150 that retain or lose activity will be of critical importance in further defining its mechanism of action.

Although the documented toxicity of 8-Aza may preclude its direct use for the suppression of HIV-1 replication in humans, the capacity of both small molecule inhibitors to suppress expression from integrated HIV-1 provirus in multiple systems serves as proof that this stage of the virus life cycle can be effectively targeted by such agents. The distinct effects of 8-Aza and 5350150, but a similar result on viral protein expression (loss of HIV-1 Gag and Env), highlight the fact that there are multiple ways of achieving a post-transcriptional block to HIV-1 gene expression. In addition, as our understanding of their mode of action increases (in particular, the host factors being affected), we will be able to refine our approach to enhance the activity towards the protein targets of interest while minimizing off target effects. Targeting this stage of the HIV-1 life cycle has the potential to greatly synergize with existing therapeutics, providing a more robust barrier to the evolution of resistance, or serve as a second-line treatment regimen in cases when first-line therapies are unsuccessful.

## SUPPLEMENTARY DATA

Supplementary Data are available at NAR Online.

## FUNDING

Ontario HIV Treatment Network; Canadian Institutes of Health Research. Funding for open access charge: Canadian Institutes of Health Research.

*Conflict of interest statement.* None declared.

## Supplementary Material

Supplementary Data
