# Supplementary material for: Characterization of novel inhibitors of HIV-1 replication that function via alteration of viral RNA processing and rev function
Source: Nucleic Acids Res. 2013 Aug 13;41(20):9471–83. doi: 10.1093/nar/gkt727 (PMC3814367; doi:10.1093/nar/gkt727)
Supplement: Supplementary Data [file supp_41_20_9471__index.html]

Characterization of novel inhibitors of HIV-1 replication that function via alteration of viral RNA processing and rev function — Characterization of novel inhibitors of HIV-1 replication that function via alteration of viral RNA processing and rev function — Supplementary Data 

# Characterization of novel inhibitors of HIV-1 replication that function via alteration of viral RNA processing and rev function

## Supplementary Data

files

**Files in this Data Supplement:**

- Supplementary Data - pdf file
