# Supplementary material for: Characterization of novel inhibitors of HIV-1 replication that function via alteration of viral RNA processing and rev function
Source: Nucleic Acids Res. 2013 Aug 13;41(20):9471–83. doi: 10.1093/nar/gkt727 (PMC3814367; doi:10.1093/nar/gkt727)

Materials and Methods

Screening of splice modulator drugs. Screening of drugs for effects on HIV-1 RNA processing was performed using the HeLa rtTA-HIV-Δ*Mls* cell line containing an inducible Tet-On HIV-1 LAI provirus (1,2) as described in our previous study (3). Activation of virus gene expression in these cells was achieved by addition of doxycycline (Dox). In drug screens, cells were seeded one day prior in IMDM containing 10% FBS, 1X Pen-Strep, and 1X Amphotericin B (Wisent Corporation) while drugs were solubilized and stored at ~1000X of its final treatment concentration in DMSO. Next, cells were treated for 4 h with 100-200 μL of drugs pre-diluted to ~25X of its final concentration in Opti-MEM (Invitrogen, #31985070) and HIV gene expression was induced with Dox (2 μg/mL). After ~24 h of compound treatment, cells and media were harvested. The effects of compounds on cell viability was assessed by a biochemical XTT assay kit (Sigma-Aldrich, #TOX2). Compounds screened were obtained from the ChemBridge Online Chemical Store (www.hit2lead.com).

Additional tests of compounds were performed using the SupT1-based cell line, 24ST1NELSG, obtained from J. Dougherty (UMDNJ-RWJMS, NJ) (4). With this cell line, latent provirus expression was induced by treatment with 1.8 uM of phorbol 12-myristate 13-acetate (PMA). Cells were treated similarly as described above with the following modifications. Approximately $1x10^6$ cells/mL of washed 24ST1NELSG cells were cultured in RPMI 1640 medium (with glutamine) containing 10% FBS, 1X Pen-Strep, and 1X Amphotericin B (Wisent Corporation). Compounds prepared for treatments were pre-diluted in RPMI medium above, incubated with cells for 4 h, then HIV-1 expression induced with 1.8 uM of PMA. After ~24 h of incubation, cells were harvested for RNA/protein or XTT assay while media or cells were lysed with 1% TX-100 for $p24^{CA}$ ELISA as described below.

Analysis of HIV-1 expression. To monitor HIV-1 gene expression, cell culture supernatants or cell lysates were assayed for production of Gag protein by a HIV-1 $p24^{CA}$ antigen capture assay kit (AIDS & Cancer Virus Program, NCI-Frederick, Frederick, MD USA). For analysis of HIV-1 by western blot, cells were solubilized in RIPA buffer, quantitated by Bradford assay, and run on 8 or 13% SDS-PAGE under reducing

conditions, and then transferred to PVDF. Normally, 25-30 μg of protein was loaded, blots blocked in either 5% Milk-T (5% skim milk, 0.05% tween-20, 1XPBS) or 3% BSA-T for 1 h at room temperature (RT) according to the antibody diluent used, and blots incubated with antibody at RT for ~2.5 h, unless otherwise specified. Specific antibodies and conditions used for Tat, anti-tubulin, and isotype-specific HRP-conjugated antibodies (Jackson ImmunoResearch) were used as described (3). Additional antibodies and conditions used in this study include a purified mouse anti-p24 supernatant from hybridoma 183: 1/300th dilution in PBS-T incubated for 1 h at RT, blocked in 3% BSA-T or 5% Milk-T for 1 h at RT. Mouse anti-gp120 purified supernatant from hybridoma 902: 1/10th dilution in PBS-T incubated normally or O/N at 4°C, blocked in 3% BSA-T at RT for 1-2.5 h. Mouse anti-Rev (Abcam, #ab85529): 1/1000th dilution in 3% BSA-T incubated normally or O/N at 4°C, loaded with 30-40 μg of protein. Rabbit anti-GAPDH (Sigma, #9545): 1/5000th dilution in 5% Milk-T incubated normally or O/N at 4°C. Generally, Western Lightning-ECL (Perkin-Elmer, #NEL101) or Western Lightning Plus-ECL (#NEL105) for anti-Rev, -Tat, and -gp120 blots was used. Signals were acquired using a ChemiDoc MP system with Image Lab software (Bio-Rad) or exposed onto autoradiography film.

Analysis of the effects of compounds on RNA processing and transport. RNA was extracted from cells by Aurum Total RNA Mini Kits (Bio-Rad, Cat. #732-6820). Purified RNA was reverse transcribed using M-MLV (Invitrogen, Cat. No. 28025-013) and resulting cDNAs were used to quantitate HIV-1 mRNA levels by qRT-PCR as described (3). The effect of drugs on HIV-1 splice site usage within the 2 kb, MS RNA class was analyzed by performing RT-PCR of cDNA obtained from RNA purified and reverse transcribed as previously described (3). For analysis of alternative splicing of cellular RNA, total RNA from three independent biological replicates of each compound treatment was reverse transcribed using random hexamers and RNaseH(-) reverse transcriptase. The samples were assayed by medium throughput RT-PCR to determine the inclusion levels of 157 alternatively spliced exons and splice sites located in 96 alternatively spliced regions of 85 genes. For this purpose we used 96 primer sets (Dataset S1), containing a fluorescently (5-FAM) labeled primer for each. The

fluorescently labeled PCR products were denatured in formamide and quantified using ABI Prism capillary sequencer (Life Technologies). The PCR reaction assembly and the subsequent liquid handling steps were carried out using 384 well PCR plates (Axygen) and automated using Biomek 2000 and Multimek 96 liquid handlers. The fragment analysis was performed on the PeakScanner software (Life Technologies) in batch mode and automated using custom scripts written in Python. The inclusion level of each exon was calculated as the amount of transcripts carrying the alternative exon relative to the total amount of all transcripts detected in the PCR reaction and results are summarized in DatasetS2.

To assess the impact of the various compounds on HIV-1 US RNA subcellular distribution, cells were treated with compounds as outlined above for 24 h then fixed in 3.7% formaldehyde, 1XPBS for 15 min., and processed as outlined by the manufacturer (Biosearch Technologies). Samples were probed with a mixture of 48 Quasar-570 5'-end labelled 50-mer oligonucleotides spanning the HIV-1 Gag coding region. Images were captured using a Leica DMR epifluorescence microscope at 630X magnification.

Effect of compounds on HIV-1 Rev subcellular distribution. HeLa Rev cells (which stably express HIV-1 Rev) were plated onto glass coverslips and treated with compounds overnight as described earlier unless otherwise indicated. For studies involving the use of import or export inhibitors, LB was added at 20 ng/ml for 2 h prior to fixing while cells treated with 4 μg/ml actinoycin D were incubated for 2 h prior to fixation. Following treatment, cells were fixed in 4% paraformaldehyde/1XPBS for 15 min. Cells were permeabilized in 1% Triton X-100/1XPBS for 5 min and blocked in 3% BSA/1XPBS solution overnight. A primary rabbit antibody against Rev was used at a 1/100th dilution for 3 h at RT or overnight at 4 C. Following washing with 1XPBS, samples were incubated with either FITC- or Cy5-labelled donkey anti-mouse antibody (Jackson laboratories) to detect immune complexes. Samples were subsequently washed with 1XPBS, mounted and viewed at 630X or 400X magnification using a Leica DMR microscope. For studies examining effects of drugs on subcellular localization of host proteins, primary antibodies used were mouse anti-hnRNP A1 (from Benoit Chabot), mouse anti-SRp20 (Invitrogen, # 334200,), or mouse anti-SC35 (Sigma, # S4045).

Statistical analysis. Data was analyzed by Microsoft Excel and expressed as means ± standard error of the mean (SEM). Differences between two groups of data (compound/drug treatment vs. DMSO (+Dox) control or compound/drug treatment vs. DMSO (+HIV) control) were compared by two-tailed Student's *t*-test. Statistical significance of results are indicated on graphs as follows: $p < 0.05$, *, $p < 0.01$, **, and $p < 0.001$, ***, unless otherwise indicated.

References

1. Zhou, X., Vink, M., Klaver, B., Verhoef, K., Marzio, G., Das, A.T. and Berkhout, B. (2006) The genetic stability of a conditional live HIV-1 variant can be improved by mutations in the Tet-On regulatory system that restrain evolution. *J Biol Chem*, **281,** 17084-17091.
2. Zhou, X., Vink, M., Berkhout, B. and Das, A.T. (2006) Modification of the Tet-On regulatory system prevents the conditional-live HIV-1 variant from losing doxycycline-control. *Retrovirology*, **3,** 82.
3. Wong, R., Balachandran, A., Mao, A.Y., Dobson, W., Gray-Owen, S. and Cochrane, A. (2011) Differential effect of CLK SR Kinases on HIV-1 gene expression: potential novel targets for therapy. *Retrovirology*, **8,** 47.
4. Micheva-Viteva, S., Pacchia, A.L., Ron, Y., Peltz, S.W. and Dougherty, J.P. (2005) Human immunodeficiency virus type 1 latency model for high-throughput screening. *Antimicrob Agents Chemother*, **49,** 5185-5188.

Supplementary Figures

Figure S1. Pattern of HIV-1 RNA splicing.

Shown at the top is the organization of the HIV-1 proviral genome indicating the position of the multiple 5' splice donor sites (SD1 to SD4) and 3' splice acceptor sites (SA1 to SA7) used in splicing of pre-mRNA. In the middle is an illustration of the alternatively spliced RNAs generated by processing of the HIV-1 genomic RNA. Indicated are the common (open boxes) and alternative exons (closed boxes) used in the generation of the SS (4 kb) and MS (1.8 kb) viral RNAs. At the bottom is a list of the nomenclature used to refer to the exon composition of the individual RNAs generated for both the SS and MS classes of HIV-1 RNAs.

Figure S2. Effects of 8-azaguanine and 5350150 on viral protein levels in the 24ST1NLESG CD4$^+$ T cell line.

24ST1NLESG cells were treated with DMSO, 10 µM of 8-Azaguanine, or 7 µM of 5350150 for 4 h, then virus gene expression induced by addition of PMA. Cells were harvested 24 h after PMA addition and cell pellets resuspended in RIPA buffer. Following fractionation on SDS-PAGE gels and transfer to PVDF membrane, blots were probed to detect HIV-1 (A) Gag, (B) Rev, or (C) Tat. Equal loading was confirmed by probing blots with anti-GAPDH or anti-tubulin (Tub.) antibody as indicated. Results shown are representative of n=3 independent assays.

Figure S3. Kinetics of 8-Azaguanine- or 5350150-induced relocalization of HIV Rev to the cytoplasm and response to leptomycin B treatment.

HeLa Rev cells were treated with (A) 45 µM of 8-Azaguanine or (B) 2 µM of 5350150 between 0-24 h (as described in Fig. 1). After indicated times, cells were fixed, stained for Rev subcellular distribution, and analyzed by microscopy. Individual cells were scored as having predominately nuclear (N>C, white), whole cell (N=C, grey), or predominately cytoplasmic (N<C, black) patterns of Rev distribution. Shown is a summary of the Rev distribution patterns for >100 cells for each time point analyzed. Graphs show data averaged from ≥ 3 independent experiments, error bars are SEM, and significant changes from control (0 h) indicated by asterisks as described in Materials and

Methods. (C) HeLa Rev cells were treated as above with either 8-azaguanine (45 μM) or 5350150 (2 μM) for 24 h. Two hours prior to fixation, leptomycin B (LB) was added to indicated samples (+LB) at a final concentration of 20 ng/ml. Localization of Rev was subsequently determined by immunofluorescence as outlined in "Materials and Methods".

Figure S4. Effect of 8-Azaguanine and 5350150 on the subcellular distribution of the nuclear factor, SC35.

HeLa Rev cells were untreated (+DMSO) or treated overnight with 45 μm of 8-Azaguanine (8-Aza) or 2 μM of 5350150 (as described in Fig. 1). Cells were subsequently fixed, permeabilized, and immunolocalized using antibodies for SC35 (αSC35) as detailed in Materials and Methods. Cells were stained with DAPI to allow imaging of the nuclei. Magnification 400X.

Figure S1 (Wong et al.)

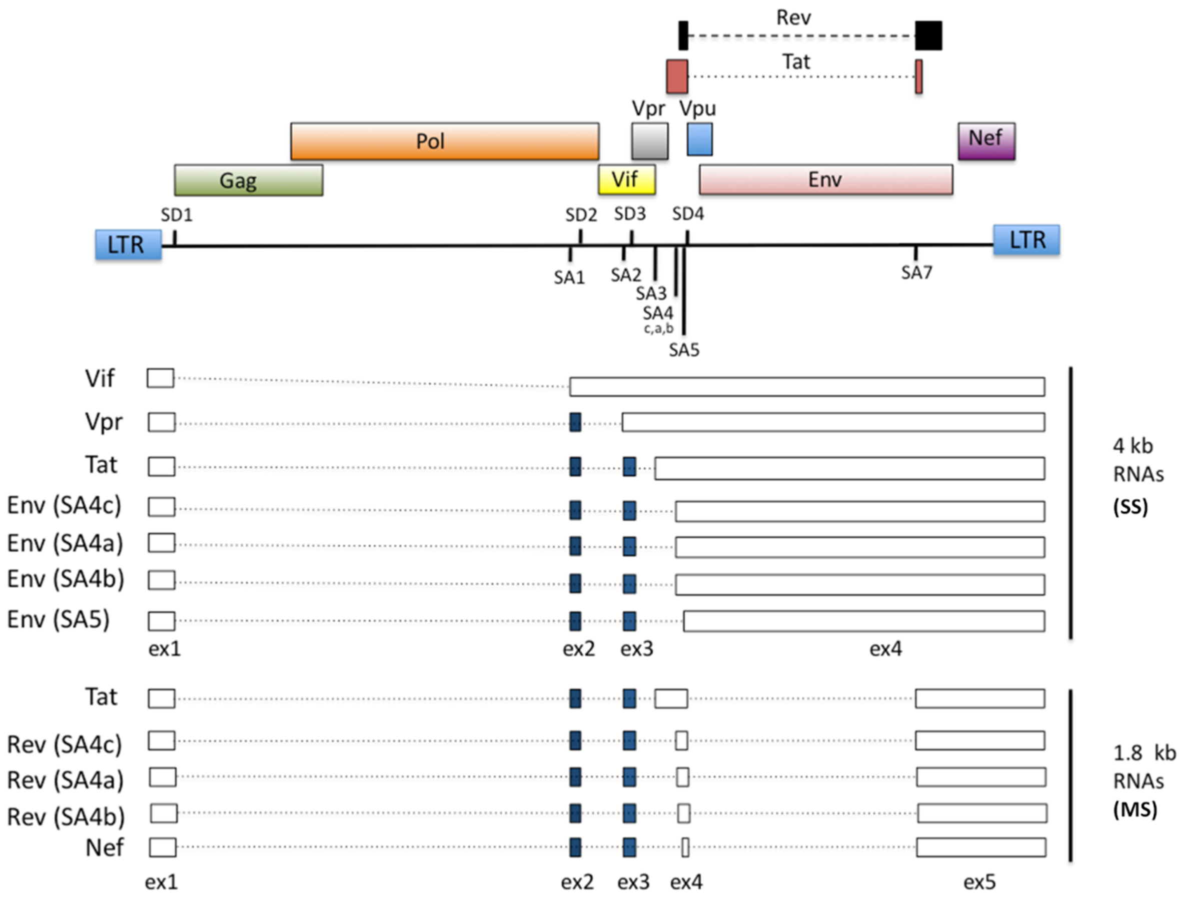

4 kb SS RNA species

vif	ex1-ex4(SA1)
vpr3	ex1-ex4(SA2)
vpr4	ex1-ex2-ex4(SA2)
tat5	ex1-ex4(SA3)
tat6	ex1-ex2-ex4(SA3)
tat7	ex1-ex3-ex4(SA3)
tat8	ex1-ex2-ex3-ex4(SA3)
env1	ex1-ex4(SA5)
env2	ex1-ex4(SA4b)
env3	ex1-ex4(SA4a)
env4	ex1-ex4(SA4c)
env5	ex1-ex2-ex4(SA5)
env6	ex1-ex2-ex4(SA4b)
env7	ex1-ex2-ex4(SA4a)
env8	ex1-ex3-ex4(SA5)
env9	ex1-ex2-ex4(SA4c)

1.8 kb MS RNA species

tat1	ex1-ex4(SA3)-ex5
tat2	ex1-ex2-ex4(SA3)-ex5
tat3	ex1-ex3-ex4(SA3)-ex5
tat4	ex1-ex2-ex3-ex4(SA3)-ex5
rev1	ex1-ex4(SA4b)-ex5
rev2	ex1-ex4(SA4a)-ex5
rev3	ex1-ex4(SA4c)-ex5
rev4	ex1-ex2-ex4(SA4b)-ex5
rev5	ex1-ex2-ex4(SA4a)-ex5
rev6	ex1-ex2-ex4(SA4c)-ex5
rev7	ex1-ex3-ex4(SA4b)-ex5
rev8	ex1-ex3-ex4(SA4a)-ex5
rev9	ex1-ex3-ex4(SA4c)-ex5
nef1	ex1-ex5
nef2	ex1-ex4 (SA5)-ex5
nef3	ex1-ex2-ex4(SA5)-ex5
nef4	ex1-ex3-ex4(SA5)-ex5
nef5	ex1-ex2-ex3-ex4(SA5)-ex5
vpr1	ex1-ex4(SA2)-ex5
vpr2	ex1-ex2-ex4(SA2)-ex5

Figure S2 (Wong et al.)

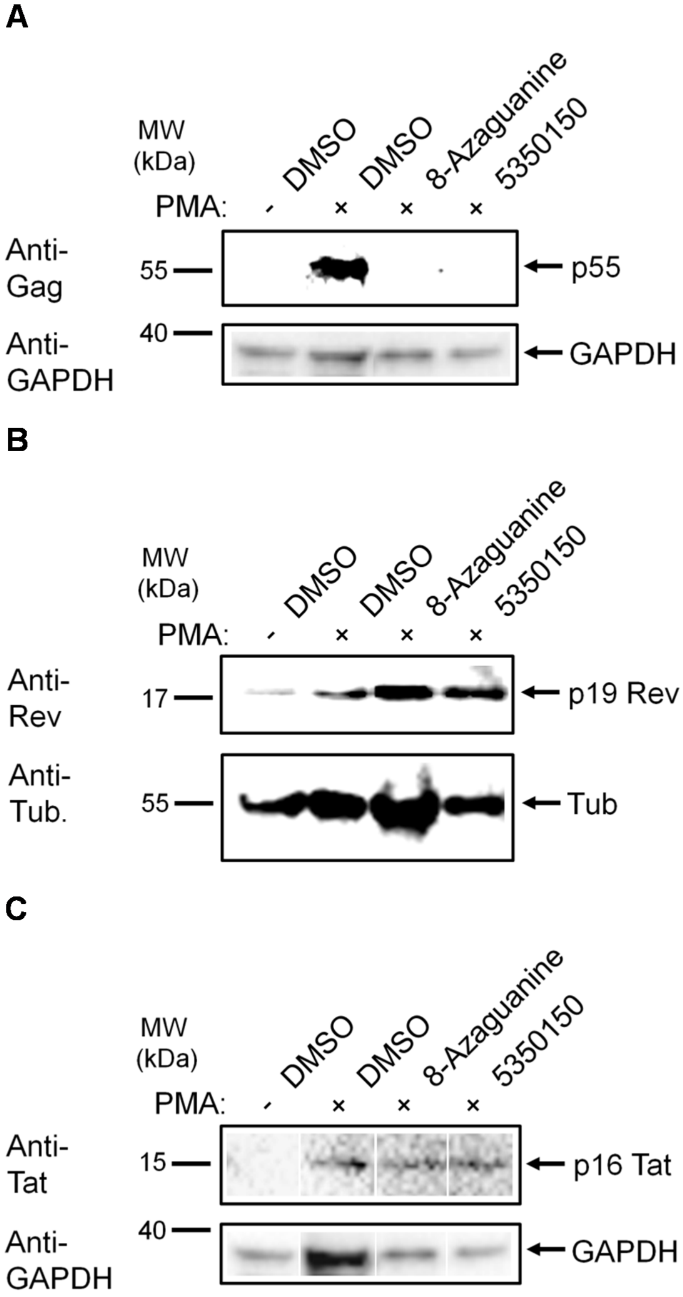

Figure S3 (Wong et al.)

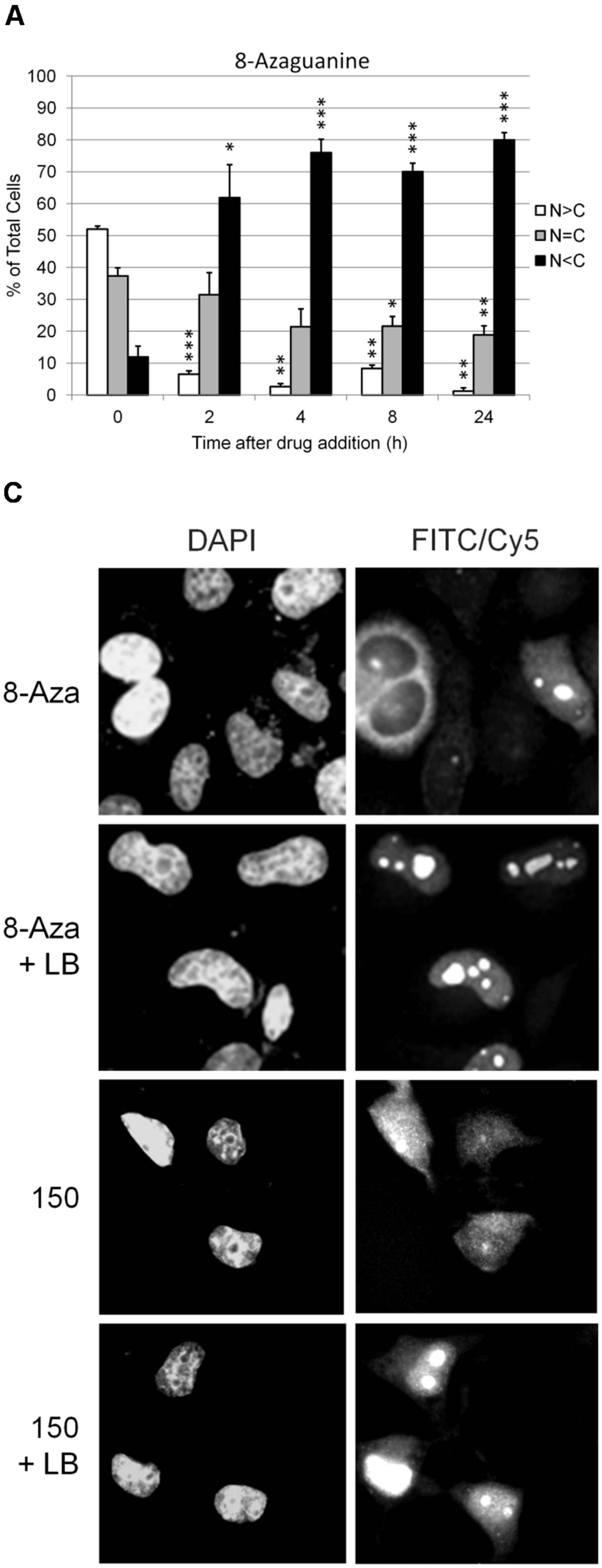

Figure S4 (Wong et al.)

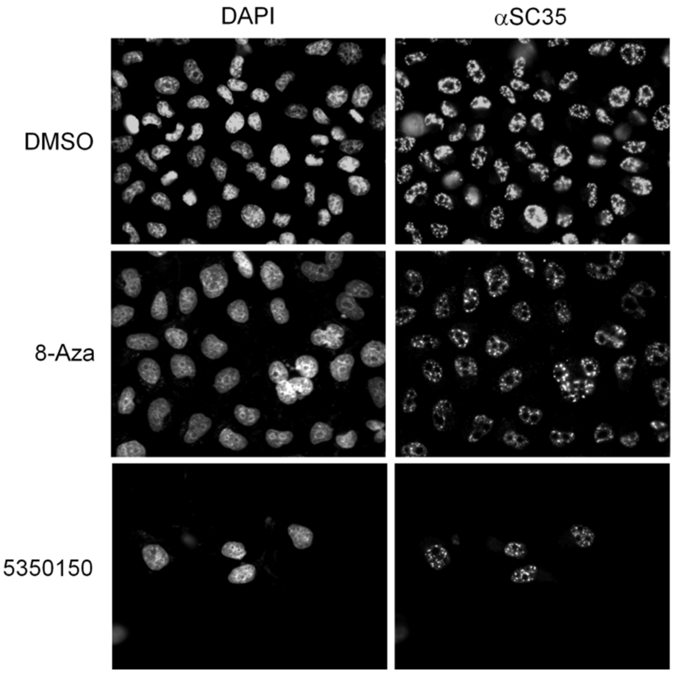

Supplement: Supplementary Data [file supp_gkt727_nar-01180-a-2013-File009.pdf]
